# Vascular remodeling 2018: the updates

**DOI:** 10.20517/2574-1209.2019.11

**Published:** 2019-04-17

**Authors:** Evgenia Gerasimovskaya, Alexander Verin

**Affiliations:** 1 Pediatric Critical Care Medicine, University of Colorado Denver, Aurora, CO 80045, USA; 2 Vasular Biology Center, Augusta University, Augusta, GA 30912, USA

## Abstract

Cardiovascular research is fundamentally important to human health, and research progress in this field could not be overemphasized. Recently we were encouraged by the editors of *Vessels Plus* to invite vascular biologists to submit their research and review articles to the special issue on “Vascular remodeling 2018: the updates” that would show up some overview of recent research from biomedical vascular science. In this special issue, we assembled five reviews and one original research paper devoted various areas of vascular biology and denoted recent advances in clinically relevant cellular and signaling mechanisms in vascular remodeling.

The review of Strassheim *et al.*^[1]^ 2018, describes the role of G proteins-coupled receptors (GPCRs) in pulmonary hypertension (PH) and potential for future targeted therapies. Pulmonary hypertension involves stiffening of pulmonary arteries and increased remodeling of small vessels. Increased pressure in the pulmonary circulation promotes hypertrophy of the heart and, eventually, cardiac failure. GPCRs signaling play a prominent role in maintaining homeostasis and dysregulated signaling contributes to cardiovascular pathology. Current therapies predominantly target vasodilatory pathways and include cGMP-protein kinase G signaling, calcium channels, endothelin receptors, and prostacyclin receptors. The pathways, by which GPCRs control transcription factors involved in vascular smooth muscle cell (VSMC) proliferation and vascular remodeling and those controlling cardiac myocyte hypertrophy or transition to cardiac failure, are far from clear. Understanding the role of GPCR-mediated signaling in PH will lead to discovering better therapies than is currently realized.

Pulmonary hypertension in neonates is (PHN) is associated with high mobility and mortality and is involved in the pathogenesis of various pediatric pulmonary disease states, such as intrauterine growth restriction and preeclampsia. Balistreri^[2]^ reviewed current view on the mechanisms of PHN and its relationship with adult PH and other adult diseases. According to the recent findings, adult PH may be result of developmental programing (DP). Vice versa, it was demonstrated that PHN could be a result of several adverse events during perinatal life. Common risk factors include drugs and alcohol abuse, high altitude living, and pollution. Importantly, that identification of specific risk factor may facilitate the development of effective therapies. Genomic imprinting is the principal driver of DP. It affects newborns primarily through maternal DNA methylation, however, other epigenetic mechanisms such as histone modifications, noncoding RNA-mediated gene silencing, and chromatin remodeling are also likely to be involved. Mechanisms and models of PHN summarized in the current review.

Epigenetic mechanisms have emerged as a one of the major drivers of vascular remodeling. Histone deacetylases (HDACs) modify core histones around DNA by removing acetyl groups from hyper-acetylated histones. More recent findings reveal that HDACs can not only deacetylate histones but many nonhistone proteins and are able to regulate numerous cellular functions such as transcription, cytoskeletal polymerization, and signal transduction. Recently, the therapeutic potential of inhibiting HDACs for the treatment of cardiovascular diseases has been appreciated. Kovacs *et al*.^[3]^ summarized the current view on the role of HDAC-mediated vascular mechanisms associated with acute lung injury (ALI) progression/preservation. ALI arises from a wide range of lung injuries such as toxins or inflammatory mediators, resulting in significant morbidity and frequently in death. A major cause of ALI is dysfunction of the pulmonary vascular endothelial barrier resulting in pulmonary infiltrates, hypoxemia and pulmonary edema. It was recently demonstrated that pharmacologic inhibition of several HDACs leads to enhancement of pulmonary vascular barrier, thereby, preventing the development of ALI. However, the mechanisms of HDAC-mediated vascular barrier preservation are ill defined. The current article provides the functional characterization of HDACs with the emphasis on their role in the regulation of endothelial barrier.

VSMC are the predominant cell type controlling large blood vessel stiffness and blood pressure. They switch between alternate phenotypes of contractile in non-pathological settings to the pathological synthetic-proliferative phenotype, associated with cardiovascular disease. In their review, Ahmed *et al.*^[4]^ 2018 focus on the role of VSMC under physiological conditions and blood vessel physiology and describe how stiffening of large arteries could be transmitted to the microcirculation of organs such as the heart and lungs. The authors describe in a simplistic manner how different molecules and structures result in the transition between contractile *vs.* synthetic-proliferative VSMC phenotype, through the mechanisms that involve of cytoskeletal proteins, myosin light chains (MLC-20, MLC-17), and myosin isoforms. They emphasize complex crosstalk between VSMCs and their surrounding matrix in healthy and in pathological conditions thus providing new insights into the mechanisms that regulate the phenotypic switch.

Thrombospondin (TSP) is a family of structurally related proteins with five distinct members (TSP1–5) that bind to surface receptors such as CD36 or the αvβ3 and αIIbβ3 integrins to regulate diverse biological processes like inflammation, immunity, control of extracellular matrix properties and composition, as well as glucose and insulin metabolism. The article of Stenina-Adognravi *et al*.^[5]^ reviews the contribution of TSPs to regulation of cancer growth and describes various functions of TSPs that link these proteins by modulating multiple physiological and pathological events that prevent or support tumor development. TSP1 and TSP2 have major role in vascular tissues, participate in platelet aggregation, and are anti-angiogenic whereas TSP4 is pro-angiogenic and pro-inflammatory in tumor models. TSP4 has shown to have a role in tumor growth and angiogenesis. TSP3 and 5 have major roles in bone development and deletion of the gene impairs skeletal development in mice. The authors summarize studies of TSP functions and roles in different systems of the organism and the complex nature of TSP interactions and functions, including their different cell- and tissue-specific effects.

The circadian clock is the cellular signaling mechanism, which controls a daily cycle of biological activities based on 24 h rhythmic oscillations. It well established that circadian clock components control cardiovascular remodeling and disruption of circadian clock genes results in alterations in rhythmic blood pressure, endothelial dysfunction thus contributing to cardiovascular diseases like atherosclerosis. The original research article of Anea *et al.*^[6]^ analyses expression of circadian clock proteins through cardiovascular tissues using immuno-histochemical approach. The studies revealed tissue-specific differences in expression and localization of circadian clock proteins in vasculature suggesting the existence of fine-tuned tissue-specific mechanisms of circadian clock regulation.
